# Prevalence of Chronic Kidney Disease and Its Association with Risk Factors in Disadvantageous Population

**DOI:** 10.1155/2012/267329

**Published:** 2012-07-09

**Authors:** Md Nurul Huda, Kazi Shahnoor Alam, Harun-Ur-Rashid

**Affiliations:** ^1^Department of Nephrology, Chittagong Medical College, Chittagong 4212, Bangladesh; ^2^Department of Nephrology, National Institute of Kidney Disease and Urology, Dhaka 1207, Bangladesh; ^3^Kidney Foundation, Dhaka 1216, Bangladesh

## Abstract

The prevalence of kidney disease, particularly diabetic and hypertensive kidney disease is increasing rapidly specially in the disadvantageous group of population throughout the world. A cross sectional survey was carried out at certain selected slum areas of Mirpur in Dhaka city of Bangladesh over the period from July 2003 to June 2005, and a total of participants ranging from 15 to 65 years were studied. The analysis discovered that 4.1% of the participants were diabetic, 11.6% were hypertensive, and 7.7% had proteinuria. Based on MDRD equation, 13.1% of the participants were detected as having chronic kidney disease (CKD) while with Cockcroft-Gault equation 16% had CKD. Accordingly, the difference between the two equations was not significant. Association of sociodemographic factors with CKD was not significant except age more than 40 years and marital status. The association between CKD and risk factors like proteinuria, obese and overweight, use of tobacco, diabetes mellitus, and hypertension was highly significant. Combined prevalence of DM, hypertension, and proteinuria among CKD group was also demonstrated to be significantly higher (3.8% with Cockcroft-Gault equation and 5.3% with MDRD equation) than that of normal population. The survey data revealed that CKD and its risk factors like DM and hypertension are alarmingly high in disadvantageous population and adding further pressure to the existing burden of CKD.

## 1. Introduction

Chronic kidney disease (CKD) is an ailment of fatality. Accordingly, many people are meeting their dooms around the world by the affliction of this disease. It is miserably factual that we barely perceive any symptom till the collapse takes place. Consequently, most of the time, it seems difficult to prevent the renal failure. 

End-stage renal disease (ESRD) has reached epidemic proportion with more than 400,000 affected individuals in the United States and well over one million worldwide [1]. This staggering number represents only the tip of the iceberg, as the incidence of chronic kidney disease (CKD) is at least 30-fold higher than that of ESRD [2]. 

The incidence of kidney disease, particularly diabetic kidney disease, is increasing rapidly in many disadvantageous populations throughout the world [3]. Disadvantageous populations are socioeconomically unprivileged having limited access to health care and low socioeconomic status related to occupational and educational level.

 CKD and the development of ESRD due to type 2 DM and systemic hypertension are particularly common in the minority population in the USA, that is, African American, Hispanic, Asian American, and native [4, 5], who are socioeconomically disadvantageous and may lack health insurance and have limited access to the health care [6]. Renal disease is also increasing, with an age-adjusted incidence of treated ESRD in Australian aboriginals approximately 20 times that of nonaboriginal people and doubling every three to four years [7]. In Sweden, low socioeconomic status related to occupational and educational level is associated with increased risk of chronic renal failure [8].

Staging of CKD is based on estimation of renal function by GFR. GFR is calculated from serum creatinine by Cockcroft-Gault and MDRD equations. Some study showed that there were a significant number of patients with normal serum creatinine level who had abnormal GFR with Cockcroft-Gault values ≤50 mL/min [9]. This group of patients may remain unrecognized by primary care physicians who rely on serum creatinine abnormality to identify renal insufficiency. 

With the increase of diabetes and hypertension, the prevalence of chronic kidney disease (CKD) is also alarmingly going up particularly in disadvantageous population. We conducted this study among the urban disadvantageous population to find out the prevalence of CKD and its association with risk factors as there was no exact data before this tiny endeavor that has been inaugurated in Bangladesh.

## 2. Materials and Methods

This cross-sectional survey was carried out at certain selected slum areas of Mirpur at Dhaka city in Bangladesh over the period from July 2003 to June 2005. A multistage clustered sampling design following a simple random sampling procedure was done to choose the study area (Mirpur slums), and a total of 1000 participants ranging from 15 to 65 years had been studied. Out of the chosen 1000 respondents, 666 were females and 334 were males.

 Inclusion criteria.
  Age between 15 and 65 years irrespective of sex.
 Exclusion criteria.
  1. Age below 15 years and above 65 years.
  2. Condition when albumin excretion is increased—exercise, pregnancy, and fever.
  3. People who did not provide consent to participate.
 
*Variables* studied were age, sex, marital status, occupation, family income, literary status, body weight (in kilogram), height (in centimeter), and body mass index (BMI) = weight in kg/height in m^2^.
 
*Risk factors* studied were diabetes mellitus, hypertension, tobacco use, overweight and obesity, and proteinuria detected by multistick.

Participants were categorized by BMI as per WHO criteria into normal (BMI 18.5–24.9), under weight (<18.5), overweight (25.0–29.9), obese (30.0–39.9), and morbid obese (≥40.00). 

Participants were considered to have diabetes mellitus if previously they had been recognized by the doctor as having DM or any documents in favour of DM or they reported taking insulin or oral antidiabetic drug or random plasma glucose ≥11.1 mmol/L with symptom. Hypertension was defined as systolic BP ≥ 140 mmHg or diastolic BP ≥ 90 mmHg or use of medication for hypertension irrespective of the blood pressure.

A random urine sample of MSU (midstream urine) had been collected from each participant using a clean catch technique and sterile container. Urinary excretion of protein and sugar was detected by multisticks named “Uripath 5” made in the UK.

Serum creatinine was measured by alkaline picrate method (Jaffe kinetic assay), which was not standardized by IDMS. Serum creatinine was determined as *μ*mol/L and converted to mg/dl by conversion factor 88.4 [10]. Ccr (creatinine clearance rate) and estimated GFR (glomerular filtration rate) were calculated from serum creatinine (mg/dL) by using Cockcroft-Gault and MDRD (modification of diet in renal disease) equations.

**Equations Developed to Predict GFR in Adult Based on Serum Creatinine**

(1) Cockcroft-Gault equation (1976)

(1) Ccr (mL/min⁡)=(140-age)×Weight(Kg)72×S. creatinine(mg/dL)×0.85  if  female.

(2) Original MDRD equations (2000) estimated GFR = 186.3 × (S. creatinine)^−1.154^ × (age)^−0.203^ × 0.742 (if female).

**Normalization of Ccr or GFR for Body Surface Area (BSA)**

Normalization of Ccr for BSA allows more accurate evaluation of renal function. Traditionally, 1.73 m^2^ is used as standard BSA. As in our population average BSA is low, it needs to be corrected by 1.73/BSA [11]:

(2) Ccr  corrected  by  BSA=Ccr(mL/min⁡)×1.73 m2BSA(m2).

Body surface area can be determined from height and weight using a monogram found in standard references:

(3) BSA(m2)=Height(cm)×weight(Kg)3600.

CKD staging was done according to K/DOQI guideline 2002. Stage 1 includes GFR ≥ 90 mL/min + proteinuria. Stage 2 includes GFR 60–89 mL/min + proteinuria. Stage 3 includes GFR 30–59 mL/mil ± proteinuria. Stage 4 includes GFR 15–29 mL/min ± proteinuria. Stage 5 includes GFR < 15 mL/min or dialysis ± proteinuria.

All the participants with CKD screened at the 1st visit were advised to have their serum checked for creatinine and urine for protein 3 months after the first check-up.

Data were collected using structured questionnaire and were finalized after field testing. Software “SPSS” and software “EpiInfo 2000” have been used for data processing and analysis. Test statistics used to analyze the data were “Chi-square test,” and “Fisher's exact test” and *P* values less than 0.05 were considered significant. There was no incentive for the slum population (respondents), and an ethical clearance was taken from departmental committee.

## 3. Observations**  **and**  **Results

**Figure 1:**
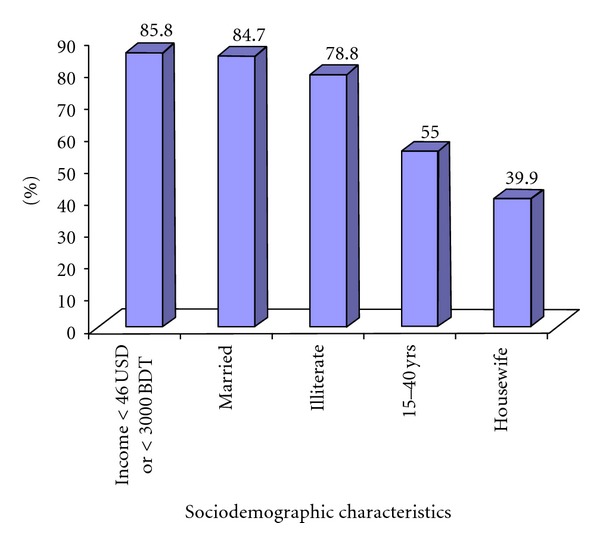
Dominant sociodemographic characteristics of the participants.

**Figure 2:**
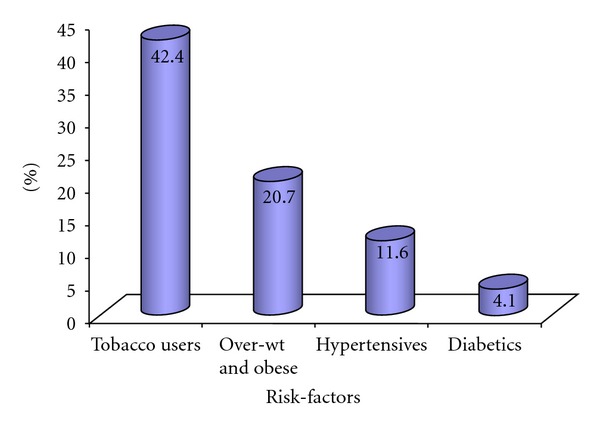
Distribution of participants by risk factors: The commonest risk-factor was use of tobacco 42.4% either as smoking or chewing or both. 11.6% was hypertensive, 4.1% was diabetics and 20.7% was over-wt and obese (BMI ≥ 25 kg/M^2^).

The analysis showed that 55% of the participants were young and early-middle-aged (from 15–40 years of age) while the mean age was 34.39 (±12.70) years. A female preponderance was observed among the participants (66.6%). The majority of the participants were married (84.7%) and illiterate (78.8%), and 85.8% had monthly income <3000 Taka (approximately USD 46) (Figure 1). In terms of occupation, the housewives comprised the main bulk (39.9%) followed by garment-workers 17.4%, small-business 9.4%, service 9%, day laborers 5.4%, rickshaw-puller 4.3%, and other jobs 10.6%. The rest of 4% was unemployed. BMI study categorized 57.5% of the participants as normal, 21.8% as underweight, 17.4% as overweight, 3% as obese, and 0.3% as morbidly obese. Out of the 1000 participants, 116 (11.6%) were hypertensive. Of them 50 (43.1%) were self-reported and 66 (56.9%) were diagnosed during the survey. Out of 41 (4.1%) diabetics, 20 (48.7%) were known and 21 (51.3%) were identified during the survey (Figure 2).

 Urine albumin analysis using multisticks demonstrated that 7.7% of the participants had proteinuria, of them 5.7% had “+” proteinuria, 1.6% had “++” and 0.4%, had “+++” proteinuria. 

Out of the total population, 1.3% of the males had serum creatinine >1.5 mg/dL and 2.7% of the female had serum creatinine >1.3 mg/dL, comprising 4% participants with raised serum creatinine. 

The mean Ccr by Cockcroft-Gault equation was 85.1 mL/minute in males and 99.2 mL/minute in females, while the mean eGFR by MDRD equation was 100.8 mL/minute in males and 133 mL/minute in females.

Out of the 1000 participiants, 13.1% were identified as CKD by MDRD equation and 16% by Cockcroft-Gault equation. Stage 3 CKD was most dominant; 10.9% in C-G equation and 6.3% in MDRD equation. The prevalence of CKD did not differ significantly between the two methods (C-G and MDRD equation) of staging (*P* = 0.687) (Table 1).

The middle-aged and elderly (from 41 to 65 years) population tend to develop CKD more than the young and early middle-aged population (from 15 to 40 years of age) (*P* < 0.001). Married groups (93.9%) also demonstrate a higher predilection to develop CKD than their unmarried counterparts (83.3%) (*P* < 0.05). Other variables were almost homogeneously distributed in both CKD and normal population (Table 2).

All the riskfactors, except BMI (overweight and obese), were present in significant proportions in CKD group compared to the normal populations (*P* < 0.001, *P* < 0.001, *P* < 0.001, *P* < 0.05, and *P* < 0.05) when Cockcroft-Gault equation was used. More than one-quarter (25.6%) of the CKD participiants were overweight and obese compared to 19.6% of those without CKD although the difference between the two groups did not reach the level of significance (*P* = 0.086) (Table 3). 

Apart from the use of tobacco, all other variables were highly prone to be associated with CKD (*P* < 0.001) when MDRD equation was used. Though use of tobacco was comparatively high in CKD group than that of normal population, the difference was not big enough to be statistically significant (*P* = 0.059) (Table 4).

## 4. Discussion

The present study conducted for “detection of chronic kidney disease and its association with risk factors in disadvantageous population” is the first ever study done in the slum area of Dhaka in Bangladesh that has urban disadvantageous population. Slum or disadvantageous population differs from other common people of Bangladesh in terms of income (average monthly income approximately USD 46), educational level (78.8% illiterate), and receiving health care facility. The prevalence study was done to examine the prevalence of diabetes mellitus, hypertension, and chronic kidney disease (based on proteinuria and low GFR) and to find the association of CKD with sociodemographic and other alleged risk factors.

We found 4.1% of the participants were diabetic and 11.6% were hypertensive in our study. The crude prevalence of type-2 DM in different communities in Bangladesh is 4.3% in rural population [12], 7.9% in urban population [13], and 8.1% in urban slums of Dhaka [14]. The prevalence of hypertension in rural population of Bangladesh with systolic blood pressure (SBP) ≥ 140 mmHg was 10.5% and with diastolic blood pressure (DBP) ≥ 90 mmHg was 9% [15] and prevalence of hypertension in urban slum population of Dhaka was 15% and 16.7%, respectively [14].

Urine protein analysis using multisticks demonstrated that 7.7% of the participants had proteinuria. Two previous population-based studies had examined the prevalence of proteinuria in adults. Iseki et al. [16] detected proteinuria that was defined by a dipstick result of trace or greater in 4 to 6% of men and 2.5 to 7% of women in a study of 1,07,192 Japanese volunteers. A similar prevalence ranging from 1% in 34–44-year-old to 6% in 55–64-year-old men was found in the US volunteers in Framingham study [17] though dipstick detection of proteinuria has better sensitivity than specificity. 

Prevalence of CKD in the present survey was 13.1% when MDRD equation was used, out of which stage 3 (GFR 30–59 mL/min) was dominant (6.3%). Prevalence of CKD using MDRD in the US adult population was 11%, out of which stage 1 was 3.3%, stage 2 3%, stage 3 4.3%, stage 4 (0.2%) and stage 5 0.2% [10].

Prevalence of CKD in this survey was 16% when Cockcroft-Gault equation was used, out of which stage 3 was 10.9%. Prevalence of CKD using C-G equation in AusDiab kidney study was also 16% [18]. The prevalence of CKD stage 3 to 5 was greater using C-G equation than MDRD equation (7% versus 4.5%) [10]. 12.9% of Japanese individuals were predicted to have CKD (using MDRD) of them stage 3 was 10.4% [19]. 

No significant difference was found in the proportion of CKD whether C-G equation or MDRD equation was used. Similar report has also been shown in NHANES III data when age of the population is less than 65–70 years. Thus the study data suggest that detection of CKD is not dependent on the method of CKD staging.

We also showed that raised serum creatinine was present in 4% of our study population in terms of sex. Kidney early evaluation program (KEEP) demonstrated that 5% of the entire KEEP population had raised serum creatinine, >1.5 mg/dL in males and >1.3 mg/dL in females [20]. 

Association of demographic factor with CKD showed that age >40 years was significantly prone to developing CKD compared to age <40 years whatever the equations were used (*P* < 0.001). In Iceland study, AusDiab kidney study, and US prevalence study 3rd National Health and Nutrition Examination survey (NHANES III) demonstrated similar results of increased CKD with increasing age in both sexes. High prevalence of stage 3 CKD among Thai individuals aged ≥35 was estimated to be about 20% by using C-G formula and about 13% by using MDRD equation [21]. The overall prevalence of CKD stages 3 to 5 in Chinese adults aged 35–74 years was lower (2.53%) when using MDRD equation [22]. 

Chronic kidney disease (CKD) was not found to be associated with sex (*P* > 0.05) in our targeted respondents. AusDiab kidney study demonstrated the risk of stage 3 to 5 CKD was greater in women (*P* = 0.002 for difference between genders). The prevalence of CKD was also higher in females (12.5%) than males (7%) in Iceland study.

In our research, CKD is more prevalent in overweight and obese population. Both males and females with low GFR (<60 mL/min) had higher BMI than that of control in the Iceland study [23]. Obesity, a component of syndrome x, explains the simultaneous increase in metabolic, cardiovascular, and renal diseases in Australian Aboriginal people [24].

The present study found that use of tobacco in the form of smoking and chewing was significantly higher in CKD group than in the normal population. Ejerblad et al. (2004) suggested that heavy cigarette smoking increased the risk of chronic renal failure for both men and women, at least CRF classified as nephrosclerosis and glomerulonephritis [25]. Among patients with DM and hypertension, smoking seems to be an independent risk factor for nephropathy, which accelerates the progression of renal failure [26–28].

In this study, diabetes mellitus and hypertension were present in significant proportions in CKD group compared to the normal population (*P* < 0.001). Combined prevalence of DM, HTN, and proteinuria in the survey in CKD group was also demonstrated to be significantly higher than that in normal population. The prevalence of CKD stage 3 to 5 was threefold higher in those with DM compared with those without DM (*P* < 0.001) [18]. The AusDiab kidney study demonstrated that reduced GFR < 60 mL/min/1.73 m^2^ was fivefold more prevalent in those with hypertension compared to those without HTN (*P* < 0.001). The association of decreased kidney function using Cockcroft-Gault equation with hypertensive and diabetic individuals was similar to decreased kidney function using the MDRD equation [10]. Two hundred diagnosed cases of DM and/or HTN were reviewed by the clinical pharmacists in primary care clinics of Columbus, OH. They demonstrated a total of 68.9% who met CKD criteria, indicating that CKD prevalence was high among the hypertensive and/or diabetic patients [29]. Besides, renal disease is also increasing in Australian aboriginals who have serious comorbidities, reflecting their poor health of them in general, including poor nutrition, infections, uncontrolled diabetes, and hypertension [30].

## 5. Conclusion 

The survey data vividly revealed that CKD is present in no less than 13% and raised serum creatinine is 4% among the urban disadvantageous population of slum area. The commonest risk factors for CKD like DM and hypertension are also alarmingly high and obviously adding to the existing burden of CKD. The association between CKD and other risk factors like age, obese and overweight, use of tobacco, DM, and HTN was also highly significant. When more than one risk factor was present, the chance of developing CKD was extensively eminent. The present study, therefore, proposes that a nationwide survey is inevitable and suggests to be conducted encompassing the entire cross-section of population to find out the prevalence of CKD and its associated risk factors, so that a preventive strategy or an entire defensive framework could be adopted or planned to reduce the disease in the community. Besides, large portion of Bangladeshi people live with extreme poverty and are alienated from the light of education. Hence, their concern about diseases is not sufficient and most of them are not capable of bearing the expenditure of treatment. Nevertheless, it is true that adopting intervention at early stages of CKD can save a family and the entire nation as well from an intense catastrophe. 

## Figures and Tables

**Table 1:** CKD staging by different equations (*N* = 1000).

Staging of CKD	Cockcroft-Gault	MDRD
**Total CKD**	**160 (16.0)** ^ ∗^	**131 (13.1)**
Stage 1	13 (1.3)	27 (2.7)
Stage 2	34 (3.4)	39 (3.9)
**Stage 3**	**109 (10.9)**	**63 (6.3)**
Stage 4	3 (0.3)	1 (0.1)
Stage 5	1 (0.1)	1 (0.1)
**Normal**	**840 (84.0)**	**869 (86.9)**

^∗^Figures in the parentheses denote corresponding %.

**Table 2:** Association between CKD (MDRD equation) and sociodemographic variables.

Socio-demographic variables	Group		*x* ^2^ value	^ ∗^ *P* values
	CKD % (*n* = 131)	Normal % (*n* = 869)		
Middle-aged and elderly (age > 40 yrs)	54.2	22.2	59.95	<0.001
Sex (female)	62.6	67.2	1.087	0.297
Occupation (housewives)	44.3	39.2	0.304	0.142
Income (≤ 2000 BDT or USD ≤ 30)	56.3	59.0	0.304	0.581
Literacy (illiterate)	77.9	78.9	0.79	0.778
Marital status (married)	93.9	83.3	9.83	0.002

^∗^Data were analysed using Chi-squared (*χ* ^2^) test, and level of significance was 0.05. *df* = 1.

**Table 3:** Association of CKD (Cockcroft-Gault equation) with risk-factors.

Risk factors	Group		*x* ^2^ value	^ ∗^ *P* values
	CKD % (*n* = 160)	Normal % (*n* = 840)		
Over-wt and obese (BMI ≥ 25 kg/M^2^)^#^	25.6	19.6	2.94	0.086
Use of tobacco^#^	58.1	39.4	19.28	<0.001
DM (self-reported + RBS > 11.1 mg/dL)^ #^	10.6	2.9	20.62	<0.001
HTN (self-reported + newly diagnosed)^#^	31.9	7.7	76.35	<0.001
Combined DM and HTN^∗^	3.8	0.6	8.71	0.004
Combined DM, HTN, and proteinuria^∗^	2.5	0.4	6.209	0.015

^#^Data were analysed using Chi-squared (*χ* ^2^) test.

^∗^Data were analysed with the help of Fisher's exact test; level of significance was 0.05.

*df* = 1.

**Table 4:** Association of CKD (MDRD Equation) with risk factors.

Risk factors	Group		*x* ^2^ value	^ ∗^ *P* values
	CKD % (*n* = 131)	Normal % (*n* = 869)		
Over-wt and obese (BMI ≥ 25 kg/M^2^)^#^	47.3	16.6	65.84	<0.001
Use of tobacco^#^	48.9	41.4	2.57	0.059
DM (self-reported + RBS > 11.1 mg/dL)^#^	16.8	2.2	61.77	<0.001
HTN (self-reported + newly diagnosed)^#^	38.9	7.5	109.81	<0.001
Combined DM and HTN^∗^	6.1	0.3	20.86	<0.001
Combined DM, HTN, and proteinuria^∗^	5.3	0	28.78	<0.001

^#^Data were analysed using Chi-squared (*χ* ^2^) test.

∗Data were analysed with the help of Fisher's exact test; level of significance was 0.05.

*df* = 1.

## Abbreviations

CKD: Chronic kidney disease
DM: Diabetes mellitus
HTN: Hypertension
BMI: Body mass index
C-G: Cockcroft-Gault formula
MDRD: Modification of diet in renal disease equation
ESRD: End-stage renal disease
Ccr: Creatinine clearance rate
GFR: Glomerular filtration rate.
